# Kv4.2 knockout mice display learning and memory deficits in the Lashley maze

**DOI:** 10.12688/f1000research.9664.2

**Published:** 2017-02-27

**Authors:** Gregory D. Smith, Nan Gao, Joaquin N. Lugo

**Affiliations:** 1 Institute of Biomedical Sciences, Baylor University, Waco, TX, 76798, USA; 2 Department of Psychology and Neuroscience, Baylor University, Waco, TX, 76798, USA

**Keywords:** Kv4.2, A type current, hippocampus, lashley maze, learning, potassium ion channel

## Abstract

**Background**: Potassium channels have been shown to be involved in neural plasticity and learning. Kv4.2 is a subunit of the A-type potassium channel. Kv4.2 channels modulate excitability in the dendrites of pyramidal neurons in the cortex and hippocampus. Deletion of Kv4.2 results in spatial learning and conditioned fear deficits; however, previous studies have only examined deletion of Kv4.2 in aversive learning tests.

**Methods**: For the current study, we used the Lashley maze as an appetitive learning test. We examined Kv4.2 wildtype (WT) and knockout (KO) mice in the Lashley maze over 4 days during adulthood. The first day consisted of habituating the mice to the maze. The mice then received five trials per day for the next 3 days. The number of errors and the time to the goal box was recorded for each trial. The goal box contained a weigh boat with an appetitive reward (gelatin with sugar). There was an intertrial interval of 15 minutes.

**Results**: We found that Kv4.2 KO mice committed more errors across the trials compared to the WT mice *p*<0.001. There was no difference in the latency to find the goal box over the period.

**Discussion**: Our finding that deletion of Kv4.2 resulted in more errors in the Lashley maze across 15 trials contribute to a growing body of evidence that Kv4.2 channels are significantly involved in learning and memory.

## Introduction

Kv4.2 is a subunit of the A–type potassium channel which mediates the excitability of pyramidal neurons in the cortex and hippocampal dendrites ^1– 3^. A-type currents regulate cell firing by attenuating action potentials and reduce excitation ^4– 8^. Kv4.2 localization in the pyramidal cell dendrites is dependent on membrane associated guanylate kinase protein (PSD-95) ^9, 10^. The highest levels of Kv4.2 are found in the CA1 of the hippocampus with less expression in the CA3 and dentate gyrus ^11^. The channels are localized to the somatodendritic regions of the hippocampal dendrites ^12– 14^. When the Kv4.2 subunit is genetically deleted, the A-type current in the CA1 pyramidal cell dendrites of the hippocampus is almost entirely removed ^15^. Disruption of the Kv4.2 has been associated with both epilepsy ^16, 17^ and autism spectrum disorder ^3^.

Kv4.2 knockout (KO) mice have a reduction in the A-type current and their threshold for long term potentiation (LTP) is also lowered, resulting in changes in synaptic plasticity ^15^. Previous research has shown the Kv4.2 KO have impaired spatial learning in the Morris water maze (MWM) and a deficit in contextual learning in fear-conditioning ^18, 19^. However, these tasks are aversive and stress could contribute to some of the learning deficits initially found. For this experiment, we used appetitive learning to examine the effects of Kv4.2 KO performance in the Lashley maze, which is a low-stress learning task that does not rely on adverse stimuli ^20, 21^.

## Materials and methods

**Animals:** The mice used for this study were Kv4.2 wildtype (WT) and KO adult males (postnatal day 60) that were generated on the 129S6/SvEv background, which had been bred for over 10 generations. All mice were bred in the Baylor University animal facility. For this study, heterozygous parents were bred to obtain both KO and WT mice and both male and female mice were used. All animals were housed in Baylor University’s animal facility on a 14 hour light 10 hour dark cycle at 22°C. Mice were all housed with sex matched littermates following weaning. All mice were given *ad libitum* access to food and water. All testing and housing complied with the National Institutes of Health Guidelines for the Care and Use of Laboratory Animals. All protocols were approved by the Baylor University Animal Care and Use Committee (Animal Assurance Number A3948-01).

**Figure 1.**
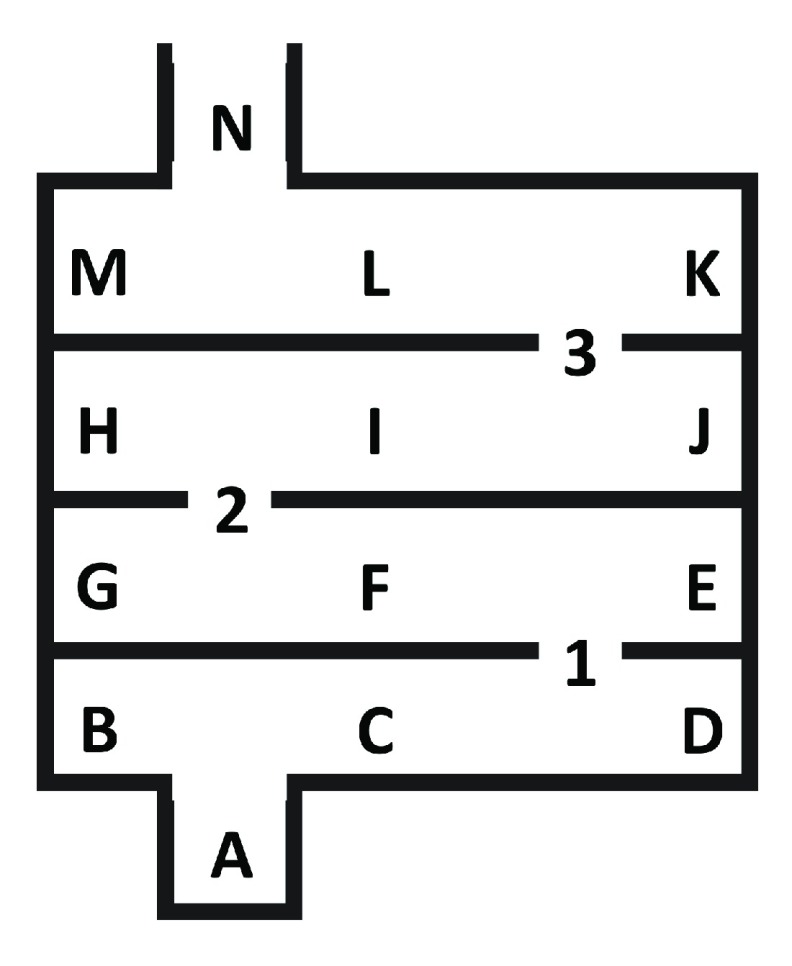
Schematic overview of the Lashley maze. The correct path: ACFILN. On day 1 the test mice were habituated to each of the chambers of the maze. For each mouse, a weigh boat containing a small amount of the gelatin was placed in the goal box (area N). The mice began habituation in section BCD with door 1 and door A blocked and were allowed to explore for 3 minutes. The mice were then moved to section GFE with doors 1 and 2 blocked, and again allowed to explore for 3 minutes. The same was then repeated in area HIJ for another 3 minutes. Finally the mice were moved to area MLK with door 3 and door N blocked and allowed to explore for 5 minutes. The apparatus was cleaned using 30% isopropanol between each mouse and a new weigh boat with fresh gelatin was used for each mouse. On day 2 a fresh weigh boat containing a small amount of gelatin was placed in the area N and the test mouse was placed in area A. The amount of time and path used to reach the goal box was recorded. The number of repeated sections the mouse entered on the way to the goal were recorded. If the mouse did not reach the end after 5 minutes it was guided to the goal using a piece of acrylic plastic used to block the doors, to prevent back tracking and wrong turns. Each mouse received 5 trials, one every 15 minutes. The same procedures were then repeated on days 3 and 4 for a total of 15 trials per mouse.

**Maze and procedure:** The details of the maze construction and procedure can be found in a previous study ^20^. The maze was constructed out of 0.25 cm thick black acrylic plastic and is 60 cm × 28 cm with 16 cm tall walls. The maze had four lanes that were evenly spaced with an additional start (area A) and goal box (area N). The start and goal boxes were 12 cm × 7.25 cm and the entrance began 12 cm from the edge of the maze. The entrance to the boxes was 6 cm wide. Doors 1, 2, and 3 were all 4 cm wide and began 12 cm from the edge of the maze. We defined an error as an entry into a dead-end cul-de-sac zone (e.g., going from arm H to zone I; Figure 1) or when the mouse travels back through a previously traveled arm of the maze (e.g., going from arm L to arm I; Figure 1). A 5% gelatin solution in double distilled water with 1.25% sucrose was prepared. The mixture was stored at 5°C until use on training and testing days. Mice were pre-exposed to the gelatin-sugar reward for 2 days prior to testing to reduce neophobia. The sugar reward was used to make the maze an appetitive test. The mice were not food deprived. The mice ate the entire reward at the end of each day. The details of the testing per day is detailed in Figure 1.

**Statistical analysis:** All statistical analyses were done using Prism 6 (GraphPad Software, La Jolla, CA), for the repeated measures, two-way ANOVAs were used to analyze these data. Separate independent t-tests were performed when an interaction was found.

## Results

**Figure 2.**
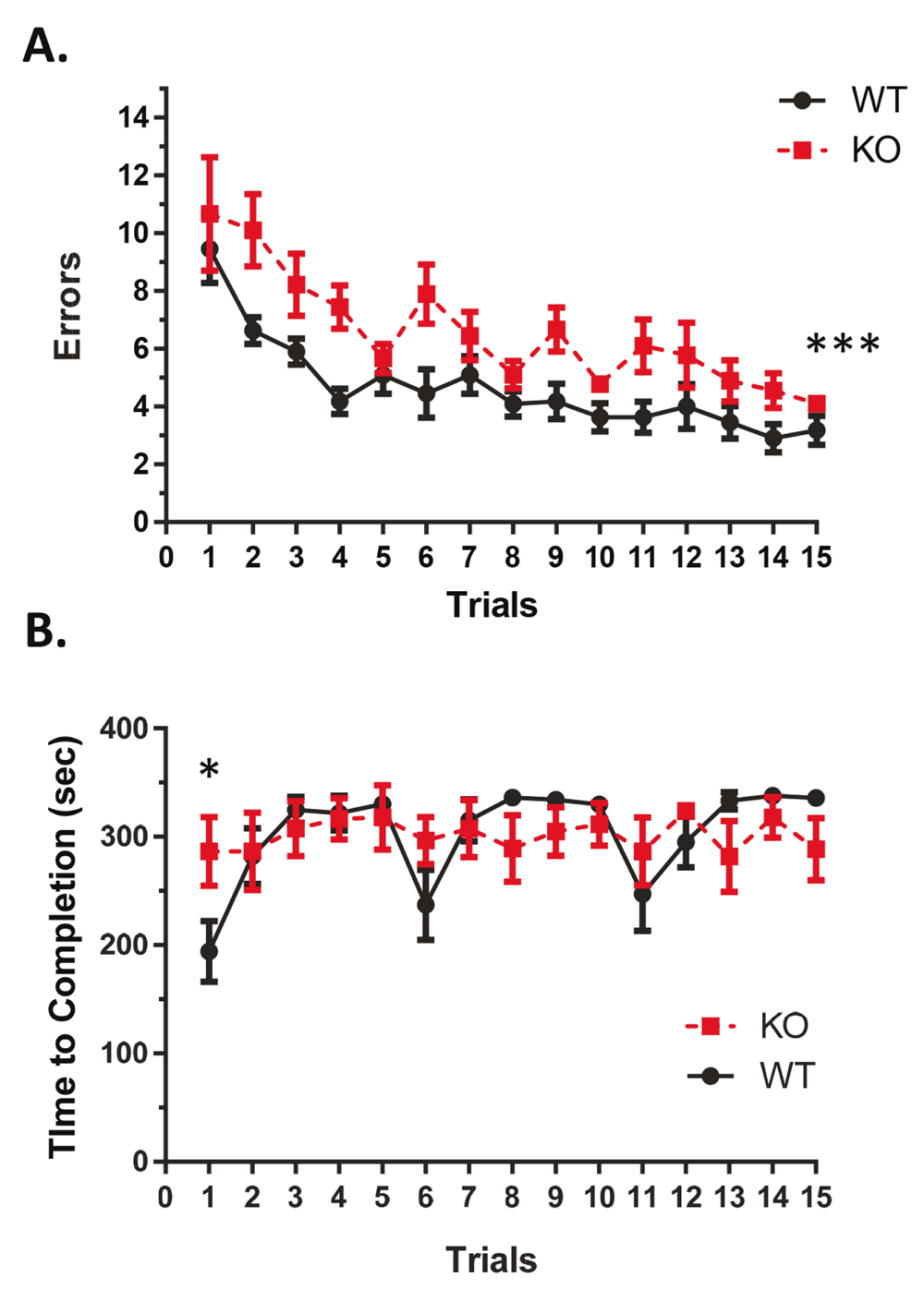
Number of errors and time to completion for maze. There was a significant difference between genotypes with the WT mice committing fewer errors when compared to the KO mice. There was no difference in the time to completion of Lashley maze for the WT and KO mice. **A**. The graph reflects the number of errors committed by the WT and KO mice across the 15 trials. **B**. The graph shows the time to completion of the Lashley maze across the 15 trials between the WT and KO mice. There was a group × time interaction across the 15 trials. An independent t-test revealed a significant difference on the first trial. No other differences were found in the remaining 14 trials. WT n = 11, KO n = 9. * = *p* < 0.05; *** = *p* < 0.001.

The WT mice committed fewer errors when compared to the KO mice in the maze over the 15 trials *F*(1, 18) = 11.9, *p*<0.001 ( Figure 2A). There was a significant effect in maze learning over the trials *F*(14, 252) = 12.9, *p* < 0.001. There was no interaction between groups over time *F*(14, 252) = 0.9, *p* = 0.52. There was no difference between Kv4.2 WT and KO mice in their time to complete the maze *F*(1, 18) = 0.01, *p* = 0.92 ( Figure 2B). There was a significant decrease for both groups in the time to find the end of the maze across trials *F*(14, 252) = 4.8, *p* < 0.001. There was a group over time interaction *F*(14, 252) = 3.1, *p* < 0.01. We ran separate t-tests over the 15 trials and found significant differences only on the first trial *t*(1,18) = 2.2, *p* < 0.05.

Data for Kv4.2 knockout mice displaying learning and memory deficits in the Lashley maze Raw data for the number of errors to find the goal box ( Figure 2A) and the duration to find the goal box ( Figure 2B) are provided in a csv file. Click here for additional data file. Copyright: © 2017 Smith GD et al. 2017 Data associated with the article are available under the terms of the Creative Commons Zero "No rights reserved" data waiver (CC0 1.0 Public domain dedication).

## Discussion

Kv4.2 wildtype and knockout mice demonstrated improvement in the Lashley maze by showing a reduction in the number of errors to find the goal box. However, the Kv4.2 KO mice committed more errors across the 15 trials compared to WT mice. By the end of the testing the Kv4.2 KO mice had similar performance to the WT mice, demonstrating that they showed improvement over the trials. The Kv4.2 KO mice showed impaired learning over the early phase of the Lashley Maze. One important consideration is that there was no difference between groups when examining the time to complete the maze. Kv4.2 KO mice required more time at the first trial, but the time to complete the maze was the same between groups for the remainder of the trials. The latency data suggest that Kv4.2 KO mice were not less active, which is in line with our previous study where we did not observe a difference in locomotor activity in the open field test ^18^. One surprising observation was that Kv4.2 WT mice had a faster latency to find the reward on the first trial. This effect was only significant on the first trial. There may be a motivation difference between the WT and KO mice for the appetitive reward. Future studies could tease out this effect.

The results from the Lashley maze complement previous studies that reported spatial learning deficits in the MWM and contextual learning deficits in the delay fear conditioning test for Kv4.2 KO mice ^18, 19^. One of the benefits of the Lashley maze is that the impact of age and sensory abilities is reduced. Impaired vision will reduce the ability of the subject to find the hidden platform in the MWM and impaired hearing can attenuate the ability of the subject to associate a tone with an aversive shock. This is important as there have been several reports that suggest ion channels may contribute to aging-related impairment ^22, 23^. Additional sensory tests would need to be performed to examine baseline sensory abilities if older subjects are used in behavioral experiments, or another approach would be to use the Lashley maze. One caveat of the Lashley Maze is that it does not tease out if the mouse is using a spatial search strategy or procedural strategy. In order to discern which type of strategy the mouse is using, complementary tests of learning could be used. The low induction of stress makes the maze a beneficial test in models that have alterations in anxiety or age-related impairments which could account for differences seen in other more aversive learning tests.

## Data availability

The data referenced by this article are under copyright with the following copyright statement: Copyright: © 2017 Smith GD et al.

Data associated with the article are available under the terms of the Creative Commons Zero "No rights reserved" data waiver (CC0 1.0 Public domain dedication). 

F1000Research: Dataset 1. Data for Kv4.2 knockout mice displaying learning and memory deficits in the Lashley maze, 10.5256/f1000research.9664.d137193 ^24^
