# Supplementary material for: Kv4.2 knockout mice display learning and memory deficits in the Lashley maze
Source: F1000Res. 2017 Feb 27;5:2456. Originally published 2016 Oct 5. [Version 2] doi: 10.12688/f1000research.9664.2 (PMC5247778; doi:10.12688/f1000research.9664.2)
Supplement: Peer review history

# Peer review: Referee response for version 2

**DOI:** 10.5256/f1000research.11889.r20575

**Published:** 2017-02-28

**Authors:** Richard E Brown

**Affiliations:** 1 Department of Psychology and Neuroscience, Dalhousie University, Halifax, NS, Canada

I have read this submission. I believe that I have an appropriate level of expertise to confirm that it is of an acceptable scientific standard.

# Peer review: Referee response for version 1

**DOI:** 10.5256/f1000research.10413.r19280

**Published:** 2017-01-13

**Authors:** Richard E Brown

**Affiliations:** 1 Department of Psychology and Neuroscience, Dalhousie University, Halifax, NS, Canada

This paper tests male Kv4.2 KO mice in the Lashley type III maze at 60+ days of age.

 It is well-written and I have only a few comments.

1. Were mice pre-exposed to the gelatin-sugar reward before the tests to prevent neophobia?
2. The figure caption for Figure 2 is garbled and should be reorganized.
3. Both groups of mice reach the same asymptote in terms of errors by day 15, so although the KO mice made more errors during training, they reached the same endpoint. What does this mean? What type of errors did they make?
4. The first trial effect in Figure 2B occurs only for the WT mice but not the KO mice. Why should this occur? What was different about the first trial each day?
5. Were mice food deprived at all?
6. How much of the reward did they eat?
7. When I look at references 14 and 15, there is the suggestion that the KO mice are slower to develop a spatial search strategy and use more procedural strategies. Is there any evidence of this in the Lashley III maze?

I have read this submission. I believe that I have an appropriate level of expertise to confirm that it is of an acceptable scientific standard.

# Peer review: Referee response for version 1

**DOI:** 10.5256/f1000research.10413.r17911

**Published:** 2016-11-24

**Authors:** Peter Backx

**Affiliations:** 1 Division of Cardiology, Peter Munk Cardiac Centre, University Health Network, Toronto, ON, Canada

Nice succinct paper.  Relevant.

 Suggested changes for the authors to consider:

- In the methods, the authors should mention how errors were identified and quantified.
- Figure 2B.  Is there any reason that the WT mice have such a pattern of in the “time to completion” data?
- The authors should provide a background on the expression patterns for Kv4.2 in mouse brains.

I have read this submission. I believe that I have an appropriate level of expertise to confirm that it is of an acceptable scientific standard.

# Response

**Published:** 2016-12-14

**Authors:** Joaquin Lugo

**Affiliations:** Baylor University, USA

We would like to thank the reviewer for their comments. We have included our replies below but will include a revised version of the manuscript when we have received both reviews. 

 Comment 1:

 We defined an error as an entry into a dead-end cul-de-sac zone (e.g., going from arm H to zone I; Fig. 1) or when the mouse travels back through a previously traveled arm of the maze (e.g., going from arm L to arm I; Fig. 1)

 Comment 2:

 We are not sure why we observed this pattern. It may be that the wildtype were more motivated on the first trial of each block. We will have to explore this behavioral change more in future studies.

 Comment 3:

 The highest levels of Kv4.2 are found in the CA1 of the hippocampus with less expression in the CA3 and dentate gyrus ^1^. The channels are localized to the somatodendritic regions of the hippocampal dendrites ^2-4^.

 References

1. Rhodes, K. J. *et al.* KChIPs and Kv4 alpha subunits as integral components of A-type potassium channels in mammalian brain. *J Neurosci* **24**, 7903-7915 (2004).
2. Sheng, M., Tsaur, M.-L., Jan, Y. N. & Jan, L. Y. Subcellular segregation of two A-type K ^+ ^channel proteins in rat central neurons. *Neuron* **9**, 271-284 (1992).
3. Maletic-Savatic, M., Lenn, N. J. & Trimmer, J. S. Differential spatiotemporal expression of K+ channel polypeptides in rat hippocampal neurons developing in situ and in vitro. *J Neurosci* **15**, 3840-3851 (1995).
4. Serodio, P. & Rudy, B. Differential expression of Kv4 K+ channel subunits mediating subthreshold transient K+ (A-type) currents in rat brain. *J Neurophysiol* **79**, 1081-1091 (1998).
